# Supplementary material for: Patterns of Intron Gain and Loss in Fungi
Source: PLoS Biol. 2004 Nov 30;2(12):e422. doi: 10.1371/journal.pbio.0020422 (PMC532390; doi:10.1371/journal.pbio.0020422)
Supplement: Table S1 — Also available at http://genes.mit.edu/NielsenEtAl/. (4.3 MB ZIP). [file pbio.0020422.st001.zip › NielsenEtAl/html/837.html]

AN8856.1.NCU06226.1.MG10185.1.FG02493.1


```
 CLUSTAL W (1.82) Multiple Sequence Alignments - Introns Inserted


Sequence 1: NCU06226.1	156 aa
Sequence 2: MG10185.1	157 aa
Sequence 3: FG02493.1	173 aa
Sequence 4: AN8856.1	153 aa
Alignment Length: 173 aa
Number Identitical Residues: 96 aa
Alignment Score (without introns) 4063


MG10185.1 	MAPKDAK--~KG1GASK--------------APKGSKQAQAASKAALKG0VH~AHKRVKA
NCU06226.1	MAPKDTK--~KG1GASK--------------AGKG-AQAKKAAQAALKG0VH~SHKKTKV
FG02493.1 	MAAKDTKGK~KA1SCSSRFCEFCTRAIADAFLTTGGNKAAQAAKAALKG0TN~SHKKVKA
AN8856.1  	MAPVQKKTG1KS~KPSD--------------------KAGAAAKAVLKG~AG0AHKTRKI
          	**. : *   *.   *.                    :*  *::*.*** .  :**  * 

MG10185.1 	RYTTSFHRPKTLQLSRAPKYPRKSVPHLPRLDEHKIIVHPLNTESAMKKIEENNTLVFIV
NCU06226.1	RYSTTFHRPKTLQLSRAPKYPRKSIPHEPRLDEHKVIVHPLNTEGALKKIEEQNTLVFIV
FG02493.1 	RYSTSFHRPKTLITSRAPKYPRRSIPHQPRLDEHKVVIHPLNTESAMKKMEENNTLVFIV
AN8856.1  	RTSTTFHRPKTLQLSRSPKYPRVSVPHLPRLDAAKIILYPLNTESAMKKIEENNTLVFIV
          	* :*:*******  **:***** *:** ****  *::::*****.*:**:**:*******

MG10185.1 	DIKANKA~QIKQALKKLYDLDVLKINTLIR2PDGQKKAYVRLTPDVDALDIAATKLALV
NCU06226.1	DVKANKA~QIKQALKKLYDIDTVKINTLIR2PDGSKKAFARLTADVDALDIAATKLGLV
FG02493.1 	DVKANKA1QIKLALKKLYDIDCLKINTLIR~PDGSKKAYARLTPDVDALDIAANKLSLV
AN8856.1  	DVKANKR~QIKAALKKLYDVETVKVNTLVR2PDGLKKAFARLTPDVDALDIAATKLAIV
          	*:****  *** *******:: :*:***:* *** ***:.***.*********.**.:*
```
